# Nothing Feels Better Than Home: Why Must Nursing‐Led Integrated Care Interventions for Older People With Chronic Conditions in Hospital‐At‐Home Be Considered?

**DOI:** 10.1111/opn.70002

**Published:** 2024-12-14

**Authors:** Rachid Akrour, Henk Verloo, Philip Larkin, Patrizia D'Amelio

**Affiliations:** ^1^ Healthcare Direction, Department of Internal Medicine, Service of Geriatrics Medicine and Geriatric Rehabilitation Lausanne University Hospital and University of Lausanne, Institute of Higher Education and Research in Healthcare Lausanne Switzerland; ^2^ Service of Old Age Psychiatry Lausanne University Hospital and University of Lausanne Prilly‐Lausanne Switzerland; ^3^ School of Health Sciences HES‐SO Valais‐Wallis Sion Switzerland; ^4^ Palliative and Supportive Care Service Lausanne University Hospital and University of Lausanne, Institute of Higher Education and Research in Healthcare Lausanne Switzerland; ^5^ Head of Service of Geriatrics Medicine and Geriatric Rehabilitation Lausanne University Hospital (CHUV) Lausanne Switzerland

**Keywords:** chronic conditions, Hospital at Home, hospital‐based home, nurse‐led clinic, older adults

Hospitalisation of older adults with a chronic conditions is associated with higher risk of nosocomial infections, delirium, falls, functional decline and even early mortality (Richardson 2006; Sharek et al. 2011; Shepperd et al. 2017; Sprivulis et al. 2006; Vasilevskis et al. 2012). Hospital‐at‐Home (HaH) interventions provide acute care treatments of a predetermined duration in the patient's home as an alternative to traditional hospital care. These interventions could shorten a hospital stay by enabling an early discharge or even becoming a complete substitution for hospital care. This would allow for continuity of acute care at home over a proscribed period of time (Gonçalves‐Bradley et al. 2017; Shepperd et al. 2016; Shepperd and Iliffe 1998). HaH interventions were developed to minimise, or even avoid, the potential iatrogenic effects of hospitalisation, improve patient and caregiver satisfaction, and reduce healthcare costs (Leong, Lim, and Lai 2021). There is growing evidence from systematic reviews demonstrating the effectiveness of HaH interventions on patient outcomes with lower mortality, reduced readmissions and lengths of stay, lower risk of long‐term care admission, lower depression and anxiety reduced costs, and improved patient satisfaction (Arsenault‐Lapierre et al. 2021; Caplan et al. 2012; Conley et al. 2016; Leong, Lim, and Lai 2021). Patients with chronic diseases who presented to emergency departments and then received HaH interventions had lower risks of readmission and long‐term admission. They also showed lower rates of depression and anxiety than patients who had received inpatient care (Arsenault‐Lapierre et al. 2021). Moreover, a meta‐analysis and a scoping review showed that patients and caregivers had positive perceptions and experiences with HaH services (Chua et al. 2022; Wang, Stewart, and Lee 2024).

Older people with chronic conditions are prone to multiple specialist follow‐ups, thereby generating significant care fragmentation (Le Couteur, Flicker, and Hilmer 2022; Sadler et al. 2023). Care fragmentation leads to adverse health outcomes and undermines patient's care experiences (Duan‐Porter et al. 2020), and contributes also to risks of medication errors (Daunt, Curtin, and O'Mahony 2023; Squires et al. 2020). Deficiencies in prioritising patient‐centred care and in the multidisciplinary continuity of care have, nevertheless, been identified (Wang, Stewart, and Lee 2024). In fact, the complex needs of an older people with multiple chronic conditions cannot be adequately addressed by a single healthcare professional; they require coordination and multidisciplinary collaboration (Araujo de Carvalho et al. 2017; Larsen, Broberger, and Petersson 2017). Thus, the World Health Organization recommends implementing integrated care models that ensure the continuity of care for older people with chronic conditions and minimise the fragmentation of care (World Health Organization 2017). Care coordination supported by nurses in an integrated healthcare model is effective for older people (Prajankett and Markaki 2021). Nurse‐led intervention models demonstrated improved patient outcomes in terms of care coordination (Gabbard et al. 2021; McParland, Johnston, and Cooper 2022), primary and secondary prevention and management of chronic diseases (Beks et al. 2023), hospital admissions (Imhof et al. 2012), emergency room visits (Counsell et al. 2007), mortality (Dorr et al. 2008), physical functioning, nutritional status and quality of life (Kasa et al. 2023).

Although recent decades have seen considerable scientific research on providing HaH interventions for older people with chronic conditions, there has been comparatively little investigation of those interventions specifically led by nurses. A literature and bibliometric search examining published articles in Medline Ovid SP up to February 21, 2024, using the search string in Appendix, comparing published studies involving HaH interventions in general and nurse‐led interventions in HaH for older adults with chronic conditions showed a lack of published research on the impact of nurse‐led interventions models in HaH (Figure 1).

**FIGURE 1:**
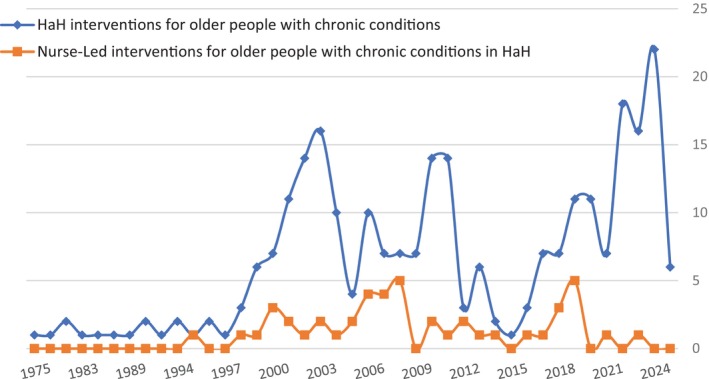
Medline database publications (since inception) examining HaH interventions and nurse‐led HaH interventions for older people with chronic conditions. Medline equations.

It is important that future nursing research should focus on highlighting the role and impact of nurse‐led interventions in HaH on the outcomes of older people and caregivers but also on health systems. Moreover, the effectiveness of nurse‐led models in this domain requires rigorous evaluation through retrospective, prospective and interventional studies. This will enhance our understanding and increase our knowledge of HaH care options that can be proposed to older people with chronic conditions. It will also give policymakers and healthcare institutions arguments for expanding efficient, home‐based, person‐centred care for this population.

## Author Contributions

R.A. contributed to the design, data analysis and writing the article. P.D., H.V. and P.L. supervised, corrected and contributed to the editing of this article.

## Conflicts of Interest

The authors declare no conflicts of interest.

## Data Availability

All data are available upon request to the authors.

## Medline Search String

1—HaH interventions for older people with chronic conditions:

(“Home Care Services, Hospital‐Based”/OR ((“hospital based” ADJ4 home) OR “hospital at home” OR “home hospital*”).ab,ti,kf.) AND (exp Aged/ OR exp. Geriatrics/ OR Geriatric Nursing/ OR multiple chronic conditions/ OR exp. comorbidity/ OR (elder* OR senior* OR geriatr* OR ((aged OR old*) ADJ2 (adult* OR patient* OR person* OR people)) OR multimorbidity or “multi‐morbidity” or “multi morbidity” or multimorbidities or “multi‐morbidities” or “multi morbidities” or multimorbid or “multi‐morbid” or “multi morbid” or comorbidity or “co‐morbidity” or “co morbidity” or comorbidities or “co‐morbidities” or “co morbidities” or comorbid or “co‐morbid” or “co morbid” or “multiple chronic conditions” or “multiple chronic illnesses” or “multiple chronic diseases” or “multiple conditions” or “multiple illnesses” or “multiple diseases” or “multiple diagnoses” or “morbidity pattern” or “morbidity patterns” or polymorbidity or “poly‐morbidity” or “poly morbidity” or polymorbidities or “poly‐morbidities” or “poly morbidities” or polypathology or “poly‐pathology” or “poly pathology” or polypathologies or “poly‐pathologies” or “poly pathologies” or pluripathology or “pluri‐pathology” or “pluri pathology” or multipathology or “multi‐pathology” or “multi pathology” or multipathologies or “multi‐pathologies” or “multi pathologies” or “multiple pathologies” or “disease cluster” or “disease clusters”).ab,ti,kf.).

2—Nurse‐led HaH interventions for older people with chronic conditions:

(“Home Care Services, Hospital‐Based”/OR ((“hospital based” ADJ4 home) OR “hospital at home” OR “home hospital*”).ab,ti,kf.) AND (exp Aged/ OR exp. Geriatrics/ OR Geriatric Nursing/ OR multiple chronic conditions/ OR exp. comorbidity/ OR (elder* OR senior* OR geriatr* OR ((aged OR old*) ADJ2 (adult* OR patient* OR person* OR people)) OR multimorbidity or “multi‐morbidity” or “multi morbidity” or multimorbidities or “multi‐morbidities” or “multi morbidities” or multimorbid or “multi‐morbid” or “multi morbid” or comorbidity or “co‐morbidity” or “co morbidity” or comorbidities or “co‐morbidities” or “co morbidities” or comorbid or “co‐morbid” or “co morbid” or “multiple chronic conditions” or “multiple chronic illnesses” or “multiple chronic diseases” or “multiple conditions” or “multiple illnesses” or “multiple diseases” or “multiple diagnoses” or “morbidity pattern” or “morbidity patterns” or polymorbidity or “poly‐morbidity” or “poly morbidity” or polymorbidities or “poly‐morbidities” or “poly morbidities” or polypathology or “poly‐pathology” or “poly pathology” or polypathologies or “poly‐pathologies” or “poly pathologies” or pluripathology or “pluri‐pathology” or “pluri pathology” or multipathology or “multi‐pathology” or “multi pathology” or multipathologies or “multi‐pathologies” or “multi pathologies” or “multiple pathologies” or “disease cluster” or “disease clusters”).ab,ti,kf.) AND (Primary Nursing/ OR Practice Patterns, Nurses'/ OR exp. Nurse Practitioners/ OR nurse specialists/ or nurse clinicians/ OR Advanced Practice Nursing/ OR exp. Nurses/ OR exp. Nursing/ OR exp. Nurse's Role/ OR exp. Nursing Care/ OR exp. Case Management/ or exp. Case Managers/ OR (apn OR apns OR acnp OR acnps OR cnss OR “specialized nurse*” OR “advanced practice nurs*” OR “advanced practitioner nurs*” OR “advanced practice registered nurs*” OR “advanced practice rn” OR “advanced practice rns” OR “advanced practitioner rn” OR “advanced practitioner rns” OR “advanced practice gerontological nurs*” OR “advanced nurs*” OR “nurse practitioner*” OR “nursing practitioner*” OR “practitioner nurse*” OR “nurse clinical practitioner*” OR “nurse specialist*” OR “nursing specialist practitioner*” OR “specialist practitioner district nurs*” OR “specialist practitioner nurs*” OR “advanced nursing” OR “consultant nurse*” OR “nurse consultant*” OR ((comanage* OR “co manage*”) ADJ3 (physician OR gp OR nurse OR nurses)) OR “primary nurse*” OR nurse‐led OR nurse‐delivered OR nurse‐coordinated OR nurse‐managed OR nurse‐driven or “nurse based intervention” or “nurse‐based intervention” or “primary nursing” or “nurse practitioner” or “nurse practitioners” or “practitioner nurse” or “practitioner nurses” or “advanced practice nurse” or “advanced practice nursing” or “advanced practice nurses” or “nurse specialist” or “nurse specialists” or “specialist nurse” or “specialist nurses” or “specialist nursing” or “nurse clinician” or “nurse clinicians” or “nurse consultant” or “nurse consultants” or “consultant nurse” or “consultant nurses” or ((“case manager” or “case‐manager” or “case management” or “case‐management”) and (nurse or nurses or nursing))).ab,ti,kf.).
